# Pulmonary arterial hypertension in hereditary hemorrhagic telangiectasia associated with *ACVRL1* mutation: a case report

**DOI:** 10.1186/s13256-022-03296-9

**Published:** 2022-03-01

**Authors:** L. J. Walsh, C. Collins, H. Ibrahim, D. M. Kerins, A. P. Brady, T. M. O Connor

**Affiliations:** 1 Department of Respiratory Medicine, Mercy University Hospital, Cork, Ireland; 2 Department of Cardiology, Mercy University Hospital, Cork, Ireland; 3 Department of Radiology, Mercy University Hospital, Cork, Ireland

**Keywords:** Hereditary hemorrhagic telangiectasia, Pulmonary arterial hypertension, Case report

## Abstract

**Introduction:**

Hereditary hemorrhagic telangiectasia is an autosomal dominant condition with an estimated prevalence of 1 in 5000. It is characterized by the presence of abnormalities of vascular structures, and may affect many organ systems, including the lungs, brain, spinal cord, gastrointestinal tract, and liver. A causative mutation is identified in approximately 97% of patients with definite hereditary hemorrhagic telangiectasia in one of three genes including a mutation in endoglin, a mutation in a locus mapped to chromosome 5, and an activin receptor-like kinase-1 (*ACVRL1*) mutation that is associated with an increased incidence of primary pulmonary hypertension. Pulmonary arterial hypertension is a rare (15–25 cases per million people) but severe vascular disorder. Heritable pulmonary arterial hypertension is associated with several gene mutations, with 75% having a mutation in the bone morphogenetic protein receptor 2 (BMPR2). However, the remaining 25% of patients have other associated genetic mutations including *ACVLR1*, which is also associated with hereditary hemorrhagic telangiectasia. Pulmonary arterial hypertension is a rare complication in patients with hereditary hemorrhagic telangiectasia (< 1% of the hereditary hemorrhagic telangiectasia population). We describe a case report with this rare occurrence.

**Case presentation:**

A 70-year-old white/caucasian Irish male presented for screening for hereditary hemorrhagic telangiectasia due to a history of recurrent epistaxis (once/week) and a family history suggestive of pulmonary hypertension. Genetic testing confirmed an *ACVRL1* mutation, while an echocardiogram and right heart catheterization confirmed pulmonary arterial hypertension. On examination, he had several mucocutaneous telangiectasia across his face. He was commenced on tadalafil and macitentan. However, this led to increased iron deficiency anemia and pedal edema. Selexipag was also added to his drug regime. He continues to require intermittent admissions for diuresis and blood transfusions.

**Conclusion:**

The association of hereditary hemorrhagic telangiectasia and pulmonary arterial hypertension is rare (< 1%). Here we describe a case of hereditary hemorrhagic telangiectasia complicated with pulmonary arterial hypertension as a result of an *ACVRL1* mutation. We also describe the clinical challenges of treating these two conditions together, as treatment options for pulmonary arterial hypertension tend to worsen hereditary hemorrhagic telangiectasia symptoms.

## Introduction

Hereditary hemorrhagic telangiectasia (HHT) is an autosomal dominant condition with an estimated prevalence of 1 in 5000 [1]. It is characterized by the presence of abnormalities of vascular structures and may affect many organ systems, including the lungs, brain, spinal cord, gastrointestinal tract, and liver [1]. Vascular abnormalities include dilation of dermal venules leading to telangiectasia, dilation of post capillary venules, and arteriovenous malformations (AVMs); these abnormalities may result in chronic bleeding, acute hemorrhage, and complications from shunting through AVMs [2–4]. The most common symptom is epistaxis from nasal mucosal telangiectasia, with approximately 90–95% of patients experiencing epistaxis by adulthood [2].

The Curacao criteria are used to establish a clinical diagnosis of HHT:

1. recurrent and spontaneous epistaxis, which may be mild to severe
2. multiple telangiectasia on the skin of the hands, lips, face, or inside of the nose or mouth
3. AVMs or telangiectasia in one or more internal organs, including the lungs, brain, liver, intestines, stomach, and spinal cord
4. A family history of HHT (first-degree relative with a clinical diagnosis of HHT or a positive genetic test) [5].

The presence of three or more criteria confers a definite diagnosis of HHT, while the presence of two criteria indicates that HHT is possible. If less than two criteria are present, a diagnosis of HHT is unlikely [5].

In 97% of patients with a definite clinical diagnosis of HHT, a causative mutation is identified in one of three genes, allowing division of HHT into three main groups:

1. HHT1 due to a mutation in endoglin, found on the long arm of chromosome 9, and is associated with a relatively higher number of pulmonary and central nervous system vascular malformations [6, 7].
2. HHT2 due to an activin receptor-like kinase-1 (*ACVRL1*) mutation, on chromosome 12, is associated with an increased incidence of primary pulmonary hypertension.
3. HHT3, which involves a locus mapped to chromosome 5 [8].

Mutations in the *SMAD4* gene have also been described in HHT. Patients with this mutation typically also have juvenile intestinal polyposis and should have regular screening colonoscopies for early identification of possible colonic malignancy [9, 10]. Both the endoglin and ALK-1 genes encode proteins that are predominantly expressed in endothelial cells. Reduced levels of these proteins are associated with impairment of blood vessel wall integrity, causing vessels to be more susceptible to dilation and remodeling during development and repair after injury [2, 6].

Pulmonary arterial hypertension (PAH) is a rare (15–25 cases per million people) but severe vascular disorder defined by an increased mean pulmonary arterial pressure (PAP) of ≥ 20 mmHg at rest, a pulmonary arterial wedge pressure (PAWP) ≤ 15 mmHg, and an increased pulmonary vascular resistance (PVR) of > 3 Wood units measured by right heart catheterization [11, 12]. Disease onset can occur at any age, but peaks in the third decade of life. PAH is a progressive condition that, if untreated, has a median survival of less than 3 years [13]. Clinical features are nonspecific and include progressive dyspnoea, decreased exercise tolerance, syncope, chest pain, edema, and fatigue, making the diagnosis challenging and often delayed [5, 11]. PAH can be associated with certain drugs (for example, anorexigens), congenital heart disease with left-to-right shunting leading to Eisenmenger syndrome, connective disease (mainly systemic sclerosis), human immunodeficiency virus, and portal hypertension [11].

Heritable PAH (HPAH) is associated with several gene mutations, with 75% having a mutation in *BMPR2*, with a 30% penetrance [14–16]. However, the remaining 25% of patients have other associated genetic mutations including *ACVLR1*, *ENG*, *SMAD4*, and *BMP9* (also known as *GDF2*), of which the first three are also associated with HHT [17, 18]. These genes all encode proteins that play a role in the transforming growth factor-beta (TGF-β) superfamily signaling pathway [11]. Overall, HHT can be complicated by HPAH, although this is a rare complication (< 1% of the HHT population) [19]. Here, we describe a case report of one gentleman with this rare occurrence. As this association is rare, we believe this case report will help expand the knowledge of this condition.

## Case presentation

A 70-year-old white/caucasian Irish male, presented for screening for HHT due to a history of recurrent epistaxis (once/week). His grandniece had tragically died suddenly aged 18 months and was found at postmortem to have severe pulmonary hypertension. Genetic testing confirmed an *ACVRL1* mutation. The child’s mother (patient’s niece) and grandfather (patient’s brother) were also found to carry this mutation, and both had clinical evidence of HHT (recurrent epistaxis and mucocutaneous telangiectasia), but echocardiography showed normal pulmonary artery pressures for both.

**Fig. 1:**
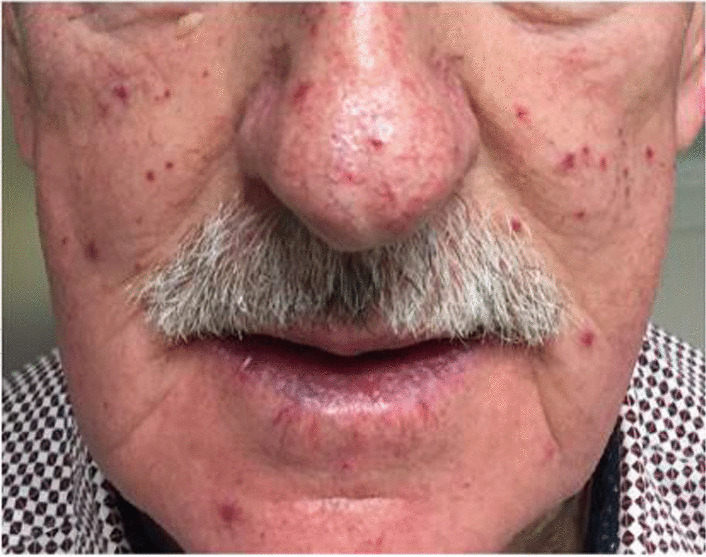
Evidence of mucocutaneous telangiectasia on face and lips of patient in question. Permission granted to share the picture. The patient gave his written informed consent for his picture to be included in this case report

Other relevant past medical history included hypercholesterolemia. He is a never smoker and was active prior to presentation. He reported that his sister had died suddenly aged 36 years while awaiting a heart and lung transplant. He has three daughters one of whom also suffers from epistaxis. They have all been referred for genetic assessment. On examination, he had several mucocutaneous telangiectasia across his face (Fig. 1). His chest was clear to auscultation. He had a loud P2 and a murmur consistent with tricuspid regurgitation.

**Fig. 2:**
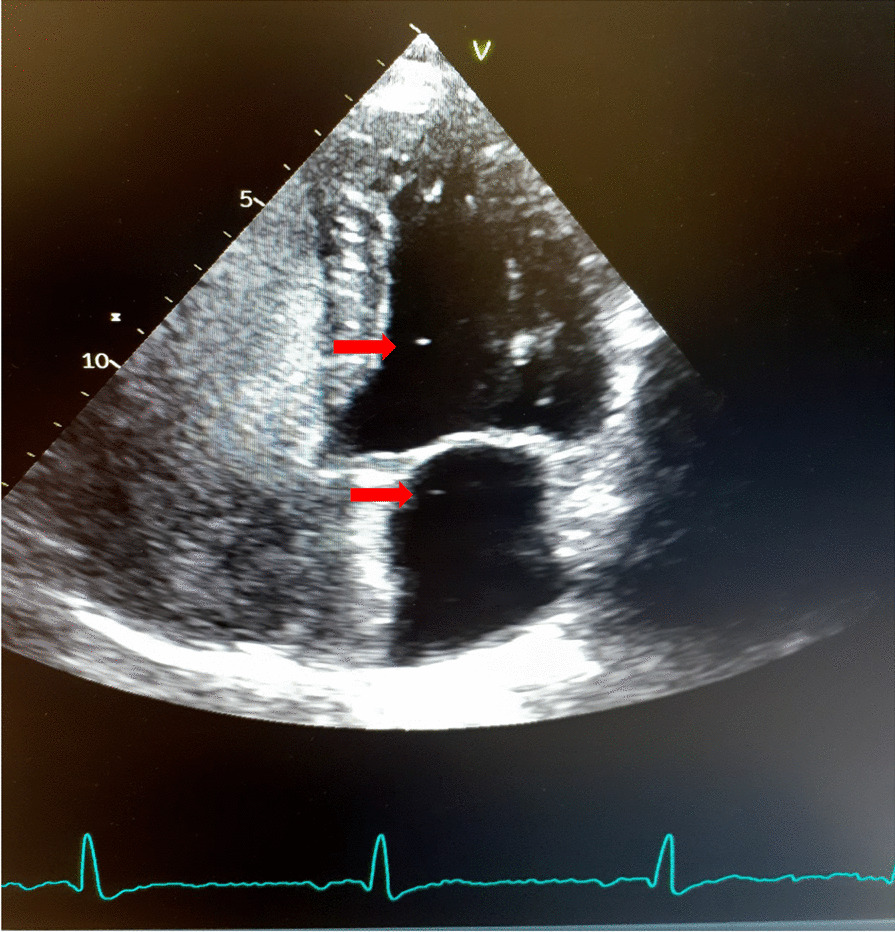
Echocardiogram (May 2019) that shows several bubbles in the left heart, eight cardiac cycles after the injection of agitated saline. This indicated that pulmonary arteriovenous malformations are likely present. Bubbles highlighted by red arrows

**Fig. 3:**
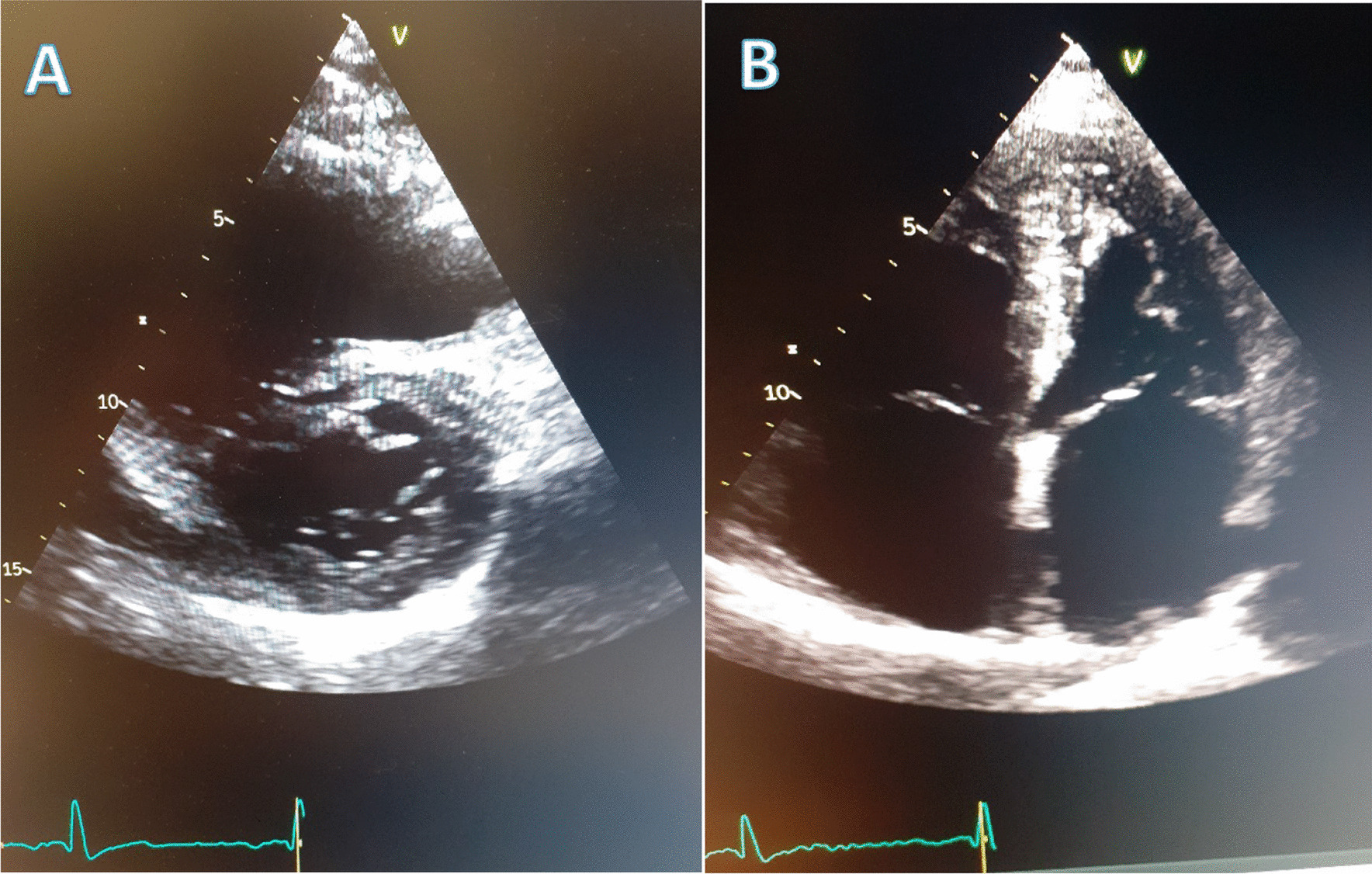
Echocardiogram evidence of right ventricular hypertrophy (**A**) and of right atrial enlargement (**B**) both consistent with underlying pulmonary arterial hypertension

An electrocardiogram demonstrated signs of right axis deviation and right ventricular hypertrophy, while an echocardiography in February 2019 showed flattening of the interventricular cardiac septum consistent with PAH. Following the intravenous administration of agitated saline (a “bubble study”), bubbles were seen in the left ventricle on the eighth cardiac cycle (Fig. 2). The late appearance is consistent with flow across an AVM rather than shunting at an interatrial or interventricular level. The estimated systolic pulmonary arterial pressure was 75–80 mmHg (normal range 18–25 mmHg) (Fig. 3).

A noncontrast computed tomography (CT) thorax showed a main pulmonary artery diameter of 3.6 cm, but no pulmonary AVMs (pAVMs), suggesting either microscopic pAVMs or shunting within other abnormal areas of the lungs accounted for the positive contrast echocardiogram. Multiple liver AVMs were demonstrated on CT thorax, with enlargement of the hepatic artery and distension of the inferior vena cava suggestive of increased venous return. CT pulmonary angiography was also preformed and ruled out chronic pulmonary embolism as a contributing factor to elevated pulmonary pressures. He was subsequently referred to a cardiologist for right heart catheterization, which confirmed severe pulmonary hypertension and elevated pulmonary vascular resistance. His pulmonary artery pressure measured at catheterization was 77/34 mmHg, wedge pressure was 4 mmHg, and pulmonary vascular resistance was 9.64 Wood units.

A brain magnetic resonance imaging (MRI) ruled out any cranial AVMs but did show increased T1 hyperintensities in the basal ganglia bilaterally. These MRI changes are said to occur in HHT patients, mainly males, and are thought to arise from manganese deposits as a result of hepatic AVMs [20].

Pulmonary function tests showed a forced vital capacity of 3.46 L (87.85% predicted), forced expiratory volume of 2.72 L (90.78% predicted), total lung volume of 7 L (100.9%), diffusing capacity of the lungs for carbon monoxide (DLCO) of 24.06 mL/min/mm (94.74% predicted), and vital capacity of 3.42 L (87.34% predicted).

Following confirmation of PAH, he was commenced on tadalafil 40 mg once a day (OD), a phosphodiesterase type 5 inhibitor, and macitentan 10 mg OD, a dual endothelin receptor antagonist. He developed a slight increase in the frequency of epistaxis thought to be secondary to tadalafil, and mild bipedal edema, thought to be secondary to macitentan, which responded to low-dose diuretic therapy. Six months after initiation of therapy for pulmonary hypertension, he began to develop symptomatic iron-deficiency anemia [hemoglobin (Hb) 9.1 g/dL], again thought to be a side-effect of macitentan, which responded to intermittent blood and iron transfusions as well as oral iron replacement. Just over a year post diagnosis, he developed worsening dyspnoea and bipedal edema and was treated with increased diuretics. To prevent further episodes of right heart failure, a prostacyclin receptor agonist, selexipag 200 mcg twice a day (BD) was added to his drug regime. His brain natriuretic protein (BNP) in May 2020 was 169 pg/mL. He continues to require intermittent admissions every 6–8 weeks for diuresis and anemia, but is otherwise symptomatically well.

## Discussion

Pulmonary arterial hypertension (PAH) is a rare but severe complication of HHT, with a prevalence of < 1% of cases [21]. *ACVRL1* mutations have been recognized to lead to a combination of HHT and PAH for several years [11]. In many of these patients, PAH was diagnosed before the clinical symptoms of HHT manifested. However, most family members of HHT patients with PAH will not develop PAH, which may indicate that additional genetic or environmental factors may be necessary to develop the HPAH phenotype [22]. PAH and HHT together have been shown to lead to worse outcomes than PAH alone, despite similar therapy and hemodynamics at time of diagnosis [22]. Compared with *BMPR2* mutation carriers and those with idiopathic PAH, *ACVRL1* mutation carriers are diagnosed at a younger age [22]. This suggests that the disease progresses more rapidly with more severe consequences. Li *et al.* [19] compared nine HHT-PAH (mutation unknown) patients to 18 idiopathic pulmonary arterial hypertension (IPAH) patients and found that 1- and 3-year survival rates were 78% and 53% for HHT-PAH patients, respectively, which was significantly lower than patients with IPAH (1- and 3-year survival 91% and 74%, *p* = 0.047).

Management of HHT and PAH together can be quite difficult as the side effects of treatment of PAH can worsen the symptoms of HHT. Results from the SERAPHIN trial showed that although macitentan was effective in improving 6-minute walk distance and reducing mortality and morbidity, there was an increase in anemia among those treated when compared with placebo [23]. Given that HHT also contributes to anemia, this can be a fine balancing act. The patient in this case did suffer greatly with worsening iron deficiency anemia since starting treatment. Furthermore, the OPTIMA study was a prospective, multicenter, single-arm, open-label phase IV study that explored the efficacy and safety of macitentan administered as initial oral combination therapy with tadalafil in newly diagnosed, treatment-naïve PAH patients. This study found that the most frequent adverse events with this drug combination were peripheral edema (28.3%), headache (23.9%), diarrhoea (19.6%), dyspnoea (15.2%), anemia (13.0%), and asthenia (13.0%) [24]. This study was in line with previously reported data [23, 25, 26]. With regards to worsening of bleeding seen with the use of macitentan in HHT and PAH patients, there is some evidence that bevacizumab can reduce the incidence of gastrointestinal bleeds [27–29]. Overall, there is a paucity of data relating to the treatment of HHT and PAH together, which makes this sensitive balancing act even more difficult.

Some of the strengths of this case report include that PAH was diagnosed on right heart catheter, which is often not the case in other reports due to the severity of patient disease [30]. This case describes the challenges involved in treatment of HHT and PAH together. A limitation of this study is that we do not have the genetic results from the patient’s daughters currently available.

## Conclusion

We present a case report of PAH and HHT occurring in a gentleman as a result of an *ACVRL1* mutation. This is a rare association in HHT patients. Furthermore, this case highlights the difficulty in treating PAH in HHT patients as treatment, such as in this case, can worsen symptoms of HHT. This case report also highlights the importance of performing an echocardiogram in patients with an *ACVRL1* mutation as this mutation is associated with a worse overall prognosis when compared with others or to IPAH.

## Data Availability

All data generated or analyzed during this study are included in this published article

## Abbreviations

*ACVRL1*: Activin receptor-like kinase-1
AVMs: Arteriovenous malformations
BMPR2: Bone morphogenetic protein receptor 2
BNP: Brain natriuretic protein
HHT: Hereditary hemorrhagic telangiectasia
HPAH: Hereditary pulmonary arterial hypertension
IPAH: Idiopathic pulmonary arterial hypertension
PAH: Pulmonary arterial hypertension
PAP: Pulmonary arterial pressure
PAWP: Pulmonary arterial wedge pressure
PVR: Pulmonary vascular resistance
TGF-β: Transforming growth factor-beta
